# NAMPT and NAPRT, Key Enzymes in NAD Salvage Synthesis Pathway, Are of Negative Prognostic Value in Colorectal Cancer

**DOI:** 10.3389/fonc.2019.00736

**Published:** 2019-08-06

**Authors:** Xiao-qin Li, Jing Lei, Lin-hong Mao, Qing-liang Wang, Feng Xu, Tao Ran, Zhi-hang Zhou, Song He

**Affiliations:** ^1^Department of Gastroenterology, The Second Affiliated Hospital of Chongqing Medical University, Chongqing, China; ^2^Department of Pathology, The Second Affiliated Hospital of Chongqing Medical University, Chongqing, China

**Keywords:** colorectal cancer, NAMPT, NAPRT, NAD, prognosis

## Abstract

Nicotinamide adenine dinucleotide (NAD) is a profoundly important cofactor in redox reactions. Nicotinamide phosphoribosyltransferase (NAMPT) and nicotinate phosphoribosyltransferase (NAPRT) are key enzymes for NAD salvage biosynthesis pathway, which reciprocally synthesize NAD to supply the main source of NAD biosythesis. However, the prognostic value of NAMPT and NAPRT in colorectal cancer (CRC) remains largely unknown. Our present study detected NAMPT and NAPRT protein expression in cancer and adjacent tissues from 261 CRC using immunohistochemical staining. We found that high expression of NAMPT or NAPRT was associated with vascular invasion, invasion depth and advanced TNM stage in CRC. High expression of NAMPT or NAPRT predicts short overall survival and disease-free survival time in CRC patients, which were further confirmed by public datasets. Furthermore, positive correlation between expression of NAMPT and NAPRT was revealed in CRC tissues and cell lines. NAPRT^high^/NAMPT^high^ patients tended to have the shortest survival time. Using the TCGA RNA-sequencing data, we showed that gene amplification, mutation, and methylation of NAPRT are more common than NAMPT. On the other hand, NAMPT gene might be targeted by more miRNAs. Finally, genes that are correlated with NAPRT or NAMPT are enriched in different pathways. In conclusion, we found that high expression of NAMPT or NAPRT predicts poor prognosis of CRC patients, but the regulatory mechanism might be distinct from each other.

## Introduction

Colorectal cancer (CRC) is the third most common malignant tumor in the world, and death from CRC accounts for 9.2% of cancer-related deaths (1). The treatment of CRC is challenging due to the complexity of its pathogenesis. Recent studies have shown that nicotinamide adenine dinucleotide (NAD) metabolism plays a major role in the progression of CRC (2). NAD, as an essential co-enzyme, mediates redox reactions in various metabolic pathways, including glycolysis, tricarboxylic acid cycle, oxidative phosphorylation, and serine biosynthesis. In addition, NAD functions as an important element for many signaling pathways by affecting the activity of several enzymes such as sirtuins (SIRTs) and poly(adenosine diphosphate [ADP]-ribose) polymerases (PARPs). Recent study revealed that NAD pool is increased in CRC tissues to reduce reactive oxygen species (3). The increased NAD also promotes the stemness maintenance and reduces the chemosensitivity of CRC cells (4). NAD anabolism can augment the aging-related secretory phenotype, which in turn promotes the occurrence of pancreatic tumors (5).

NAD can be synthesized through the *de novo* and salvage pathways. Even though most cells can synthesize NAD through the *de novo* pathway from tryptophan, the main source of NAD is supplied by salvage pathways that synthesize NAD from nicotinamide (NAM) or nicotinic acid (NA) as precursors because of its high efficiency. In the classical salvage pathway, NAMPT as a rate-limiting enzyme, convert NAM to nicotinamide mononucleotide (NMN), which is then adenylated to NAD by nicotinamide mononucleotide adenylyl transferase (NMNATs) (2). In the Preiss-Handler pathway, another kind of salvage pathway, nicotinate phosphoribosyltransferase (NAPRT) transforms NA to nicotinic acid mononucleotide (NAMN), which is then conjugated with ATP to generate NAD (6). NAMPT has been reported to be highly expressed in various tumors, such as gastric cancer, breast cancer, pancreatic cancer and prostate cancer, to promote tumor cell glycolysis, proliferation, survival, invasion, metastasis and chemotherapy resistance (7–10). In addition, NAPRT was also reported to be highly expressed in various cancers including ovarian cancer and pancreatic cancer (11). Silencing of NAPRT decreases NAD level and increases chemosensitivity of cancer cells. NAMPT and NAPRT have been reported to reciprocally synthesize NAD. As mentioned above, silencing of NAPRT or inhibition of NAPRT by 2-hydroxynicotinic acid increased the sensitivity to FK866, a specific inhibitor of NAMPT. More recently, selective cytotoxicity of FK866 to mesenchymal gastric cancer cells has also been reported due to loss of NAPRT expression (7). Up to now, the prognostic value of NAPRT in CRC patients has yet to be revealed, and the prognostic significance of NAMPT in CRC needs validation in large sample size although high expression of NAMPT has been reported to predict worse prognosis in only 35 cases (12).

Our present study aimed to elucidate the prognostic value of NAMPT and NAPRT in 261 CRC patients. We showed that the high expression of NAMPT or NAPRT is associated with vascular invasion, deep invasion and advanced TNM stage. Both NAMPT and NAPRT were of negative prognostic value for CRC patients. Moreover, NAMPT expression was positively correlated with NAPRT in CRC tissues and cell lines. NAPRT^high^/NAMPT^high^ patients tended to have shortest overall survival and disease-free survival time. Using the public sequencing data, we revealed that amplification of NAPRT gene and methylation of NAPRT promoter were more frequent in CRC than NAMPT, but there are more miRNAs that might bind with NAMPT genes. Furthermore, the NAPRT-correlated genes are enriched in Oxidative phosphorylation and Metabolic pathways, while NAMPT-correlated ones are enriched in TNF signaling pathway, PI3K-Akt signaling, NOD-like receptor signaling, Ubiquitin mediated proteolysis. In conclusion, high expression of NAMPT and NAPRT predict poor prognosis of CRC patients, which implies that combined inhibition of NAMPT and NAPRT might be effective for CRC therapy.

## Materials and Methods

### Patient Selection and Primary Tissue Samples

Surgical specimens of colorectal cancer, a total of 261 cases, from November 2012 to 2015 were collected at the Second Affiliated Hospital of Chongqing Medical University. The cancer tissues and corresponding adjacent tissues are applied to produce a human tissue microarray (TMA). The criteria for selecting patients were as follows: (1) No other types of tumors were combined; (2) No radiotherapy or chemotherapy before surgery.

The study was approved by the research ethics committee at the Second Affiliated Hospital of Chongqing Medical University ((2019)133). In addition, the research obtained the informed consent of the patients.

### Immunohistochemistry

The tissue microarray was used for immunohistochemical staining as previously described (13). Briefly, the paraffin sections of TMA were dewaxed in a 65°C incubator for 1 h. Then they were deparaffinized in xylene and rehydrated through a graded ethanol series. The sections were placed in phosphate-buffered saline (PBS) and heated in a pressure cooker for 5 min for antigen retrieval. They were then washed with a PBS buffer shaker. A 3% hydrogen peroxide solution was added to the sections for 20 min at room temperature to eliminate endogenous peroxidase, and then washed with PBS. The primary antibodies against NAPRT (Proteintech, catalog:13549-1-AP, China, 1:100) and NAMPT (Proteintech, catalog:11776-1-AP, China, 1:100) were incubated overnight at 4°C, then incubated with horseradish peroxidase (HRP)-conjugated secondary antibodies (ZSGB-BIO, catalog:PV-9000, China) for 1 h at 37°C incubator. Sections were stained with diaminobenzidine (BOSTER, catalog:AR1022, China) and then counterstained with hematoxylin. Sections incubated with PBS were used as negative control.

### Evaluation of Immunostaining Results

Evaluation of NAPRT and NAMPT staining included staining intensity (classified as: 0, no staining; 1, weak staining; 2, moderate staining; and 3, strong staining) and the proportion of positive cancer cells (classified as: 0, <5, 1, 5–25, 2, 26–50, 3, 51–75, and 4, 76–100%) (12). In the statistical analysis, the two above described values were multiplied to obtain the composite expression score (CES), ranging from 0 to 12. According to the median CES, patients were divided into low and high expression of NAPRT/NAMPT. The results were independently performed by two pathologists.

### Bioinfomatic Analysis

The expression of NAMPT/NAPRT, the methylation level of their promoters, and the correlated gene sets in CRC tissues were determined by UALCAN^1^ (14), which downloaded the level-3 TCGA RNA-sequencing data from 31 cancer types from the TCGA website for further analysis. The expression of NAMPT or NAPRT was analyzed in cancer samples from 288 colon cancer patients and 166 rectal cancer patients, 10 rectum tissues and 41 colon tissues. Transcripts per million (TPM) expression value was used as the measure of expression. The tumor stage information of 12 colon cancer patients and 10 rectal cancer patients was not available. The miRNAs which potentially bind with the 3′-UTR region of NAMPT/NAPRT were predicted with TargetScan^2^. The prognostic value of NAMPT/NAPRT in CRC was tested by PROGgnesV2^3^ (15), which compiled data from public repositories such as GEO, EBI Array Express and TCGA. Data sets GSE24551 (Exon microarray in 160 CRCs) and GSE30378 (Exon microarray in 95 CRCs) were used for analyzing the prognostic value of NAPRT in CRC and datasets GSE17536 (Gene expression array in 174 CRCs) and GSE39582 (Affymetrix mRNA expression profiles in 572 colon cancer patients) were used for analyzing the prognostic value of NAMPT in CRC. The mutation or amplification of NAMPT/NAPRT was validated with cBioportal^4^, which also used TCGA data. Three CRC TCGA datasets with gene mutation information were involved: TCGA Nature 2012 (*n* = 276), TCGA pancancer (*n* = 594), and TCGA provisional (*n* = 640). Promoter methylation was evaluated by UALCAN using MeDIP-seq data. The Beta value indicates level of DNA methylation ranging from 0 (unmethylated) to 1 (fully methylated).

### Cell Culture

The human CRC cell line HT29, HCT116, SW480 and LoVo and normal intestinal epithelial NCM460 cells were purchased from the American Type Culture Collection. The cells were cultured in Dulbecco's Modified Eagle Medium (DMEM; Gibco, Carlsbad, CA), add 10% fetal bovine serum (FBS; Gibco, Carlsbad, CA), 2 U/mL penicillin-streptomycin, vitamins, 1 mmol/L sodium pyruvate, 2 mmol/LL-glutamine, and non-essential amino acids (Thermo Scientific, Watertown, MA) in a 37°C in 5% CO_2_.

### Western Blot Analysis

The Proteins samples were collected using radio immunoprecipitation assay (RIPA) buffer (Beyotime, Shanghai, China) according to the manufacture's protocol. After gel electrophoresis and transforming proteins to PVDF membranes, PVDF membranes were blocked in 5% milk/TBS-T for 2 h at 37°C. Then, PVDF membranes incubation with antibodies against NAPRT (Proteintech, catalog:13549-1-AP, China, 1:1,000), NAMPT (Proteintech, catalog:11776-1-AP, China, 1:100) and GAPDH (ZSGB-BIO, catalog:TA-08, China, 1:1,000) overnight at 4°C, respectively. The membranes were then washed, followed by incubation with respective horseradish peroxidase-conjugated secondary antibodies for 2 h. At last, protein bands were visualized using the SuperSignal West Pico maximum sensitivity substrate (Pierce, Rockford, IL, USA).

### Statistical Analysis

All statistical analyses used the Statistical Package for the Social Sciences (SPSS) software, version 20 to perform. Patient characteristics were compared using a combination of frequency distribution analysis and descriptive statistics. The survival curve is plotted by the Kaplan-Meier method. Cases with distant metastasis were removed when it comes to DFS. We used the Chi-square test or Fisher's exact test for categorical variables. Univariate analysis was performed using the Cox proportional hazards model to test for independent significance by eliminating unimportant explanatory variables backwards. It was considered statistically significant unless the *P* < 0.05.

## Results

### High Expression of NAPRT Is Correlated With Advanced TNM Stage and Poor Prognosis of CRC Patients

**Figure 1:**
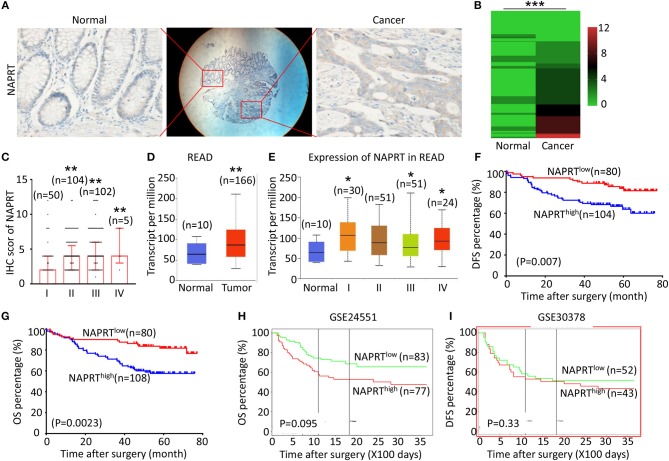
High NAPRT expression is correlated with advanced TNM stage and poor prognosis of CRC patients. **(A)** The typical images showing the expression of NAPRT in CRC tissue and adjacent tissues. **(B)** Heat map showing the IHC scores of NAPRT in CRC tissues and corresponding normal intestinal epithelium. **(C)** IHC scores of NAPRT in CRC tissues with different TNM stages. **(D)** UALCAN analysis showing the mRNA expression of NAPRT in rectal cancer and normal intestinal epithelium. **(E)** UALCAN analysis showing the mRNA expression of NAPRT in different stages of rectal cancer. **(F)** The Kaplan–Meier survival analysis showing DFS of CRC patients with high or low NAPRT expression level. **(G)** The Kaplan–Meier survival analysis showing OS of CRC patients with high or low NAPRT expression level. **(H)** The Kaplan–Meier survival analysis showing that OS of CRC patients with high or low NAPRT mRNA expression from GSE24551. **(I)** The Kaplan–Meier survival analysis showing that OS of CRC patients with high or low NAPRT mRNA expression from GSE30378.COAD, colon adenocarcinoma; READ, rectal adenocarcinoma; *P < 0.05; **P < 0.01; ***P < 0.001.

We tested the expression of NAPRT in 261 CRC patients, including 153 males and 108 females. The details of clinicopathological features of patients in the study are shown in Table 1. The representative immunohistochemical images are shown in Figure 1A. Data from the Human Protein Atlas, which maps all the human proteins in cells, tissues and organs, showed that the protein abundance of NAPRT is moderate in normal colon and rectum (Supplementary Figure 1A) and CRC tissues (Supplementary Figure 1B). We found that the expression of NAPRT in CRC tissues was dramatically higher than adjacent tissues (Figure 1B), which increased as TNM stage advanced (Figure 1C). UALCAN analysis also revealed the same results (Figures 1D,E). Chi-square test revealed that high NAPRT expression significantly correlated with vascular invasion (*P* = 0.001), higher T-stage (*P* < 0.001), lymphnode metastasis (*P* = 0.008) and advanced TNM stage (*P* < 0.001) in CRC (Table 1). We got follow-up data from 188 patients among the 261 patients. Kaplan-Meier survival rate indicates that high expression of NAPRT is associated with shorter disease-free survival (DFS) (Figure 1F) and overall survival (OS) (Figure 1G) in CRC patients. Data from GSE24551 and GSE30378 also showed that patients with high NAPRT expression tend to have short OS (Figures 1H,I). Univariate Cox regression analysis showed that high NAPRT expression was prognostic risk factor for DFS (hazard ratio (HR) = 2.765, 95% confidence interval (CI) = 1.468–5.210, *P* = 0.002) and OS (HR = 2.622, 95% CI = 1.459–4.713, *P* = 0.001) (Table 2). These results indicate that high NAPRT expression is correlated with advanced TNM stage and poor prognosis of CRC patients.

**Table 1:** Correlation between NAPRT/NAMPT expression and clinicopathological features in cancer tissues from 261 CRC patients.

**Features**	**No. of patients (%)**	**NAPRT expression status**		***P***	**NAMPT expression status**		***P***
		**Low (*n* = 120) No. patient (%)**	**High (*n* = 141) No. patient (%)**		**Low (*n* = 169) No. patient (%)**	**High (*n* = 92) No. patient (%)**	
Gender							
Male	153 (58.6)	77 (50.3)	76 (49.7)	0.093	104 (68.0)	49 (32.0)	0.195
Female	108 (41.4)	43 (39.8)	65 (60.2)		65 (60.2)	43 (39.8)	
Age							
≤ 60	131 (50.2)	62 (47.3)	69 (52.7)	0.660	84 (64.1)	47 (35.9)	0.831
>60	130 (49.8)	58 (44.6)	72 (55.4)		85 (65.4)	45 (34.6)	
Tumor size (cm)							
≤ 4.0	142 (54.4)	73 (51.4)	69 (48.6)	0.054	95 (66.9)	47 (33.1)	0.427
>4.0	119 (45.6)	47 (39.5)	72 (60.5)		74 (62.2)	45 (37.8)	
Differentiation degree							
Well	94 (36.0)	42 (44.7)	52 (55.3)	0.693	56 (59.6)	38 (40.4)	0.411
Moderate	98 (37.6)	45 (45.9)	53 (54.1)		67 (68.4)	31 (31.6)	
Poor	69 (26.4)	33 (47.8)	36 (52.2)		46 (66.7)	23 (33.3)	
Vascular invasion							
Negative	162 (62.1)	88 (54.3)	74 (45.7)	**0.001**	122 (75.3)	40 (24.7)	**0.000**
Positive	99 (37.9)	32 (32.3)	67 (67.7)		47 (47.5)	52 (52.5)	
Nerve invasion							
Negative	204 (78.2)	99 (48.5)	105 (51.5)	0.118	140 (68.6)	64 (31.4)	**0.013**
Positive	57 (21.8)	21 (36.8)	36 (63.2)		29 (50.9)	28 (49.1)	
T-stage							
T1 + T2	57 (21.8)	42 (73.7)	15 (26.3)	**0.000**	47 (82.5)	10 (17.5)	**0.002**
T3 + T4	204 (78.2)	78 (38.2)	126 (61.8)		122 (59.8)	82 (40.2)	
N stage							
0	156 (60.0)	84 (53.8)	72 (46.2)	**0.008**	124 (79.5)	32 (20.5)	**0.000**
I	65 (25.0)	23 (35.4)	42 (64.6)		30 (46.2)	35 (53.8)	
II	40 (15.0)	13 (32.5)	27 (67.5)		15 (37.5)	25 (62.5)	
M stage							
0	256 (98.1)	119 (46.5)	137 (53.5)	0.378	167 (65.2)	89 (34.8)	0.349
I	5 (1.9)	1 (20.0)	4 (80.0)		2 (40.0)	3 (60.0)	
TNM stage							
I	50 (19.2)	36 (72.0)	14 (28.0)	**0.000**	43 (86.0)	7 (14.0)	**0.000**
II	104 (39.8)	47 ((34)45.2)	57 (54.8)		79 (76.0)	25 (24.0)	
III	102 (39.1)	36 (35.3)	66 (64.7)		45 (44.1)	57 (55.9)	
IV	5 (1.9)	1 (20.0)	4 (80.0)		2 (40.0)	3 (60.0)	
NAMPT expression							
≤ 4	169 (64.8)	100 (59.2)	69 (40.8)	**0.000**	169 (100.0)	0 (0.0)	**0.000**
>4	92 (35.2)	20 (21.7)	72 (78.3)		0 (0.0)	92 (100.0)	

*Chi-square test was used to evaluate the correlation between NAPRT/NAMPT expression and clinicopathological features. The bold values indicated that the P-value was smaller than 0.05*.

**Table 2:** Univariate Cox regression analysis of the risk factors in colorectal cancer.

**Clinicopathologic features**	**OS**			**DFS**		
	**HR**	**95% CI**	***P***	**HR**	**95% CI**	***P***
Gender (male/female)	0.869	0.518–1.457	0.594	1.028	0.591–1.789	0.923
Age (≤60/>60)	0.915	0.550–1.522	0.733	0.709	0.404–1.244	0.230
Tumor size (cm) (>4.3/ ≤ 4.3)	1.596	0.959–2.655	0.072	1.456	0.840–2.524	0.181
Differentiation degree (well/moderate + poor)	0.901	0.530–1.532	0.700	1.054	0.600–1.850	0.855
T-stage (T3 + T4/T1 + T2)	9.112	2.224–37.343	**0.002**	15.330	2.117–111.036	**0.007**
N-stage (N1 + N2/N0)	4.047	2.359–6.944	**0.000**	3.369	1.902–5.967	**0.000**
M-stage (M1/M0)	6.456	2.304–18.902	**0.000**	7.305	2.246–23.760	**0.001**
Nerve invasion (positive/negative)	2.838	1.674–4.811	**0.000**	2.442	1.358–4.390	**0.003**
Vascular invasion (positive/negative)	2.015	1.210–3.355	**0.007**	1.995	1.148–3.465	**0.014**
TNM stage (III + IV/I + II)	4.332	2.507–7.487	**0.000**	3.333	1.881–5.905	**0.000**
NAPRT (high/low)	2.622	1.459–4.713	**0.001**	2.765	1.468–5.210	**0.002**
NAMPT (high/low)	3.518	2.085–5.935	**0.000**	3.05	1.743–5.337	**0.000**

*OS, overall survival; DFS, disease- free survival. The bold values indicated that the P-value was smaller than 0.05*.

### High Expression of NAMPT Is Correlated With Advanced TNM Stage and Poor Prognosis of CRC Patients

**Figure 2:**
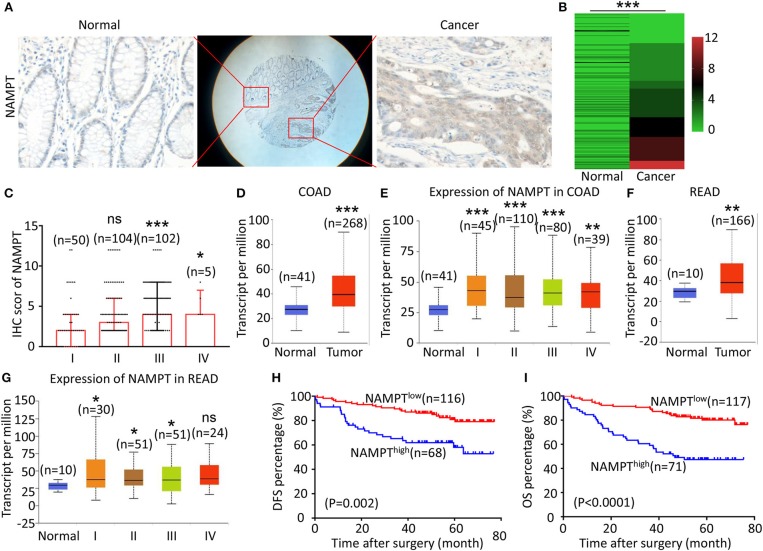
High NAMPT expression is correlated with advanced TNM stage and poor prognosis of CRC patients. **(A)** The typical images showing the expression of NAMPT in CRC tissue and adjacent tissues. **(B)** Heat map showing the IHC scores of NAMPT in CRC tissues and corresponding normal intestinal epithelium. **(C)** IHC scores of NAMPT in CRC tissues with different TNM stages. **(D)** UALCAN analysis showing the mRNA expression of NAMPT in colon cancer and normal intestinal epithelium. **(E)** UALCAN analysis showing the mRNA expression of NAPRT in different stages of colon cancer. **(F)** UALCAN analysis showing the mRNA expression of NAMPT in rectal cancer and normal intestinal epithelium. **(G)** UALCAN analysis showing the mRNA expression of NAPRT in different stages of rectal cancer. **(H)** The Kaplan–Meier survival analysis showing DFS of CRC patients with high or low NAPRT expression level. **(I)** The Kaplan–Meier survival analysis showing OS of CRC patients with high or low NAPRT expression level. COAD, colon adenocarcinoma; READ, rectal adenocarcinoma; *P < 0.05; **P < 0.01; ***P < 0.001.

The representative immunohistochemical images of NAMPT staining are shown in Figure 2A. Data from the Human Protein Atlas showed that NAMPT is moderately expressed in normal colon and rectum (Supplementary Figure 2A) and CRC tissues (Supplementary Figure 2B). We found that NAMPT in CRC was highly expressed in CRC tissues compared with adjacent tissues (Figure 2B), which increased as TNM stage advanced (Figure 2C). UALCAN analysis also revealed the same results in both colon cancer (Figures 2D,E) and rectal cancer (Figures 2F,G). High NAMPT expression significantly correlated with vascular invasion (*P* < 0.001), nerve invasion (*P* = 0.013), higher T-stage (*P* = 0.002), lymphnode metastasis (*P* < 0.001), and advanced TNM stage (*P* < 0.001) in CRC (Table 1). Kaplan-Meier survival rate indicates that high expression of NAMPT is associated with shorter disease-free survival (DFS) (Figure 2H) and overall survival (OS) (Figure 2I) in CRC patients. Data from GSE17536 (*n* = 174) and GSE39582 (*n* = 572) also showed that patients with high NAMPT expression tend to have short OS (Supplementary Figures 3A,B). Univariate Cox regression analysis showed that high NAMPT expression was prognostic risk factor for OS (HR = 3.518, 95% CI = 2.085–5.935, *P* < 0.001) and DFS (HR = 3.05, 95% CI = 1.743–5.337, *P* < 0.001) (Table 2). These results indicate that high NAMPT expression is correlated with advanced TNM stage and poor prognosis of CRC patients.

### Positive Association Between the Expression of NAMPT and NAPRT in CRC Tissues

**Figure 3:**
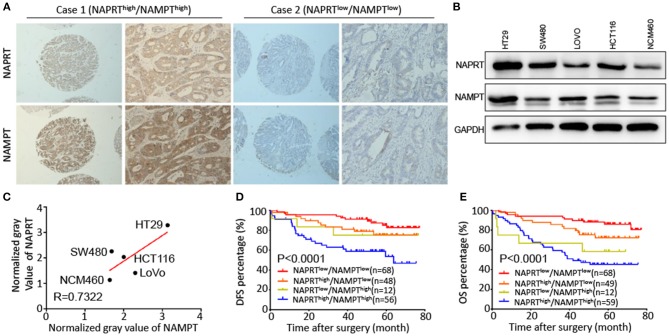
Positive association between the expression of NAMPT and NAPRT in CRC tissues. **(A)** Representative images show high expression of both NAPRT and NAMPT (case 1), as well as low expression of NAPRT and NAMPT (case 2). **(B)** Western bot showing the expression of NAPRT and NAMPT in normal intestinal epithelia NCM460 and CRC cell line HT29, HCT116, SW480, and LoVo cells. **(C)** Gray values detected from western blot results. **(D,E)** The Kaplan–Meier survival analysis showing that CRC patients with high expression of NAPRT and NAMPT tend to have a shorter DFS **(D)** or OS **(E)** time.

Both NAPRT and NAMPT are important enzymes in the salvage NAD biosynthesis pathway. We found that the NAMPT protein abundance was positively correlated with NAPRT in CRC tissues by immunohistochemical staining (Table 1). Figure 3A shows CRC tissues with high expression of both NAPRT and NAMPT (case 1), and low expression of both NAPRT and NAMPT (case 2) simultaneously. We detected the expression of these two proteins in normal intestinal epithelia NCM460 and CRC cell line HT29, HCT116, SW480, and LoVo by western blot (Figure 3B). The results revealed positive correlation between the expression of NAPRT and NAMPT (Figure 3C). Kaplan-Meier survival showed that patients with high expression of NAPRT and NAMPT had the lowest cumulative OS and DFS, and those with low expression of NAPRT and NAMPT have the best prognosis in CRC (Figures 3D,E). These results revealed the positive association between the expression of NAMPT and NAPRT in CRC.

### Bioinformatic Analysis of the Potential Regulatory Mechanism of NAMPT/NAPRT Expression

**Figure 4:**
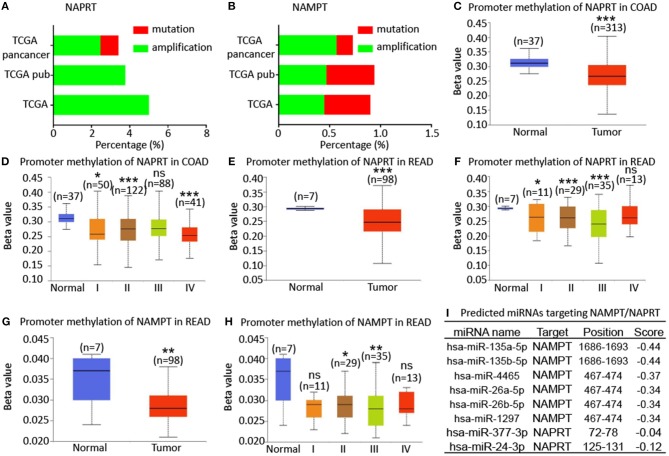
Bioinformatic analysis of the potential regulatory mechanism of NAMPT/NAPRT expression. **(A)** Mutation and amplification analysis of NAPRT gene in CRC via cBioportal. **(B)** Mutation and amplification analysis of NAMPT gene in CRC via cBioportal. **(C)** Promoter methylation of NAPRT in colon cancer using UALCAN. **(D)** Promoter methylation of NAPRT gene in different stages of colon cancer. **(E)** Promoter methylation of NAPRT in rectal cancer using UALCAN. **(F)** Promoter methylation of NAPRT gene in different stages of rectal cancer. **(G)** Promoter methylation of NAMPT gene in rectal cancer. **(H)** Promoter methylation of NAPRT in different stages of rectal cancer using UALCAN. **(I)** Predicted miRNAs that might bind with 3′-UTR of NAPRT/NAMPT gene by TargetScan. COAD, colon adenocarcinoma; READ, rectal adenocarcinoma; *P < 0.05; **P < 0.01; ***P < 0.001.

To explore the potential regulatory mechanism of NAMPT/NAPRT expression, we firstly analyzed the mutation and gene amplification of these two genes in CRC via cBioportal. The results showed that NAPRT gene is amplified in 2.47–5.0% CRC samples (Figure 4A), while the number for NAMPT gene is only 0.45–0.57% (Figure 4B). We then analyzed the promoter methylation level using UALCAN. The Beta value indicates level of DNA methylation ranging from 0 (unmethylated) to 1 (fully methylated). We found that promoter methylation of NAPRT gene is significantly decreased in colon cancer (Figure 4C). Furthermore, promoter methylation of NAPRT gene decreases as tumor advances (Figure 4D). Similar results were found in rectal cancer (Figures 4E,F). Although the methylation of NAMPT promoter is lower in CRC tissues than adjacent tissues (Figures 4G,H), the overall promoter methylation of NAMPT is dramatically low in comparison with NAPRT. We then predicted the miRNAs that might bind with 3′-UTR of NAPRT/NAMPT gene by TargetScan. There are 16 miRNAs that could bind with NAMPT gene, and only 2 conserved miRNAs for NAPRT (Figure 4I). Taken together, these results implied that gene amplification and promoter methylation is the main cause for NAPRT up-regulation, while NAMPT is mainly regulated by miRNAs.

### KEGG Pathway Analysis of Genes Associated With NAMPT/NAPRT Expression in CRC

**Figure 5:**
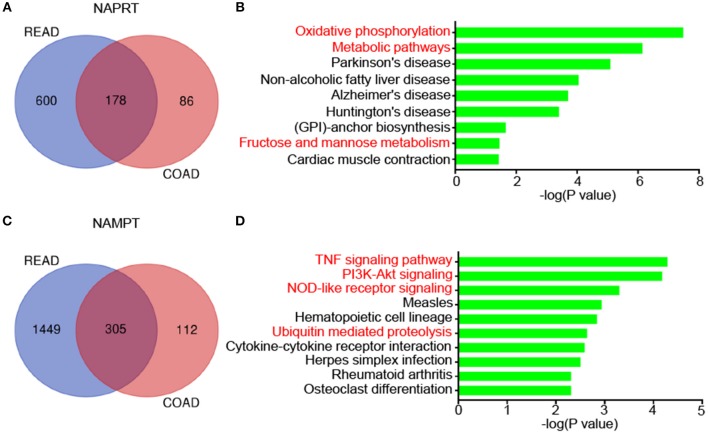
KEGG pathway analysis of genes associated with NAMPT/NAPRT expression in CRC. **(A)** The 178 genes that significantly correlated with NAPRT expression in both colon cancer and rectal cancer. **(B)** KEGG analysis of the correlated genes with NAPRT. **(C)** The 305 genes that significantly correlated with NAMPT expression in both colon cancer and rectal cancer. **(D)** KEGG analysis of the correlated genes with NAMPT.

We then revealed the genes that correlate with NAPRT/NAMPT expression in colon cancer and rectal cancer using UALCAN. There are 178 genes that significantly correlated with NAPRT expression in both colon cancer and rectal cancer (Figure 5A). KEGG analysis showed that these genes are enriched in pathways such as Oxidative phosphorylation, Metabolic pathways, Parkinson's disease, Non-alcoholic fatty liver disease (Figure 5B). There are 305 genes that significantly correlated with NAMPT expression in both colon cancer and rectal cancer (Figure 5C). In contrast with NAPRT, KEGG analysis showed these NAMPT-correlated genes are enriched in pathways such as TNF signaling pathway, PI3K-Akt signaling, NOD-like receptor signaling, Ubiquitin mediated proteolysis (Figure 5D). These results suggest that NAPRT and NAMPT might have different regulatory mechanism.

## Discussion

The prognosis of CRC patients is still not satisfying, although much progress has been made in the last decades. Better understanding of the pathological and molecular features of CRC is urgent for developing novel targets for the intervention of CRC and improving the prognosis. Our present study found that NAPRT and NAMPT were both highly expressed in CRC tissues, and correlate with vascular invasion, higher T-stage, lymph node metastasis and advanced TNM stage. At the same time, both of them predicted poor outcomes of CRC patients. Metabolic reprogramming is one of the hallmarks of tumors (16), which ensures that tumor cells overcome oxygen and nutrient deficiencies and maintain the unlimited proliferation potential (17). Tumor cells tend to get their energy from aerobic glycolysis. In this process, NAD can promote tumor cells proliferation by enhancing glycolysis. NAMPT and NAPRT are the two key enzymes in NAD salvage biosynthesis pathway, both of which are essential in the regulation of metabolism and signal transduction in caner (18). Although nicotinamide riboside kinase 1 (NRK1) also plays an important role in NAD salvage pathways converting NR to NMN (19), the prognostic value of NRK1 in tumor needs to be revealed.

The molecular mechanisms regulating the expression of NAMPT and NAPRT are still poorly known. Using public sequencing data, we revealed that the cause of NAMPT/NAPRT expression might be distinct from each other. Amplification of NAPRT gene and methylation of NAPRT promoter are dramatically more frequent in CRC than NAMPT, but there are more miRNAs that might bind with NAMPT genes. These results implied that gene amplification and promoter methylation might be the main causes for NAPRT up-regulation, while NAMPT mainly regulated by miRNAs. It has been reported that promoter hypermethylation led to loss of NAPRT expression in most cancer types, with the frequencies ranging from 5 to 65% (20). Chowdhry et al. (21) recently reported that cancers arose from normal tissue with highly expressed NAPRT have a high frequency of NAPRT genes amplification. The survival of these cancers completely and irreversibly depends on NAPRT. By comparison, tumors originated from normal tissues with low NAPRT expression depend on NAMPT for survival. Some studies demonstrated that NAMPT was targeted by miRNAs such as miR-135a (22), miR-22 (23), miR-300 (24), miR-206 (25), miR-182 (26), miR-26a (27), miR-374a (28), miR-26b (29), and miR-34a (30). Moreover, long non-coding RNAs such as lncRNA-GACAT3and SIRT1 AS-lncRNA could also regulated the expression of NAMPT (22, 23). In a latest study, a new AS-lncRNA RP11-22N19.2, which was transcribed from the antisense strand of NAMPT, has been reported to enhance the transcriptional activity of NAMPT in triple negative breast cancer (8). In addition, the transcription of NAMPT gene can be directly regulated by FOXO1 in breast cancer (31). Mutant IDH1 can also downregulate NAPRT in glioma (32). Although most studies showed negative association between promoter methylation and gene expression, the correlation between NAPRT promoter methylation and NAPRT expression needs further validation. We further found the pathways correlated with NAMPT or NAPRT are significantly different. The NAPRT-correlated genes are enriched in metabolic pathways, while NAMPT-correlated ones are enriched in classical signaling pathways. Nevertheless, how NAMPT and NAPRT play their oncogenic roles in CRC remains to be explored.

The present study confirmed positive correlation between NAMPT and NAPRT expression CRC tissues and cells for the first time. A study by Lee et al. (7) reported that the low expression level of NAPRT predicts high efficiency of FK866, NAMPT inhibitor, in gastric cancer. Loss of NAPRT in cancer cells make cancer cells sensitive to NAMPT inhibitor FK866 and possibly also allow increasing the therapeutic efficiency of FK866 by coadministration of NA analog (33). Therefore, we suppose that co-administration of NAMPT inhibitor and NAPRT inhibitor may effectively decrease NAD levels and kill cancer cells. It is reasonable to speculate the over-expression of these proteins might promote tumor cell growth and diffusion to the maximum extent, which needs to be validated in future work. Moreover, whether there is regulatory effect between NAPRT and NAMPT needs to be demonstrated in the future.

In conclusion, we first investigated the clinicopathological and prognostic value of NAMPT and NAPRT in cancer and adjacent tissues from 261 CRC patients. Both NAMPT and NAPRT are highly expressed in CRC tissues, but the regulatory mechanism might be distinct. High expression of NAMPT and NAPRT are associated with poor prognosis of patients with CRC. These results suggest that NAMPT and NAPRT might be novel markers for the diagnosis and treatment of CRC.

## Data Availability

The raw data supporting the conclusions of this manuscript will be made available by the authors, without undue reservation, to any qualified researcher.

## Ethics Statement

The research ethics committee at the Second Affiliated Hospital of Chongqing Medical University ((2019)133).

## Author Contributions

The research was conceived and designed by SH and ZZ. The experiments were carried out by XL and JL. The data was analyzed by QW and LM. The clinical specimens were collected by FX and TR. The manuscript was written by XL and JL.

### Conflict of Interest Statement

The authors declare that the research was conducted in the absence of any commercial or financial relationships that could be construed as a potential conflict of interest.

## Footnotes

^1^http://ualcan.path.uab.edu/

^2^http://www.targetscan.org/

^3^http://genomics.jefferson.edu/proggene

^4^http://www.cbioportal.org/
