# Supplementary material for: NAMPT and NAPRT, Key Enzymes in NAD Salvage Synthesis Pathway, Are of Negative Prognostic Value in Colorectal Cancer
Source: Front Oncol. 2019 Aug 6;9:736. doi: 10.3389/fonc.2019.00736 (PMC6691178; doi:10.3389/fonc.2019.00736)
Supplement: Supplementary file 1 [file Table_1.DOC]

**Supplementary Material**

**Supplementary Figure 1**. The expression of NAPRT in normal tissues and cancer tissues. (A) The protein abundance of NAPRT in normal tissues from the Human Protein Atlas. (B) The protein abundance of NAPRT in cancer tissues from the Human Protein Atlas.

**Supplementary Figure 2**. The expression of NAMPT in normal tissues and cancer tissues. (A) The protein abundance of NAMPT in normal tissues from the Human Protein Atlas. (B) The protein abundance of NAMPT in cancer tissues from the Human Protein

**Supplementary Figure 3**. High expression of NAMPT is associated with short OS in CRC patients. (A) The Kaplan–Meier survival analysis showing that OS of CRC patients with high or low NAMPT mRNA expression from GSE17536. (B) The Kaplan–Meier survival analysis showing that DFS of CRC patients with high or low NAMPT mRNA expression from GSE39582.*, P<0.05;**,p<0.01;***,P<0.001
