# Supplementary figures and images for: NAMPT and NAPRT, Key Enzymes in NAD Salvage Synthesis Pathway, Are of Negative Prognostic Value in Colorectal Cancer
Source: Front Oncol. 2019 Aug 6;9:736. doi: 10.3389/fonc.2019.00736 (PMC6691178; doi:10.3389/fonc.2019.00736)

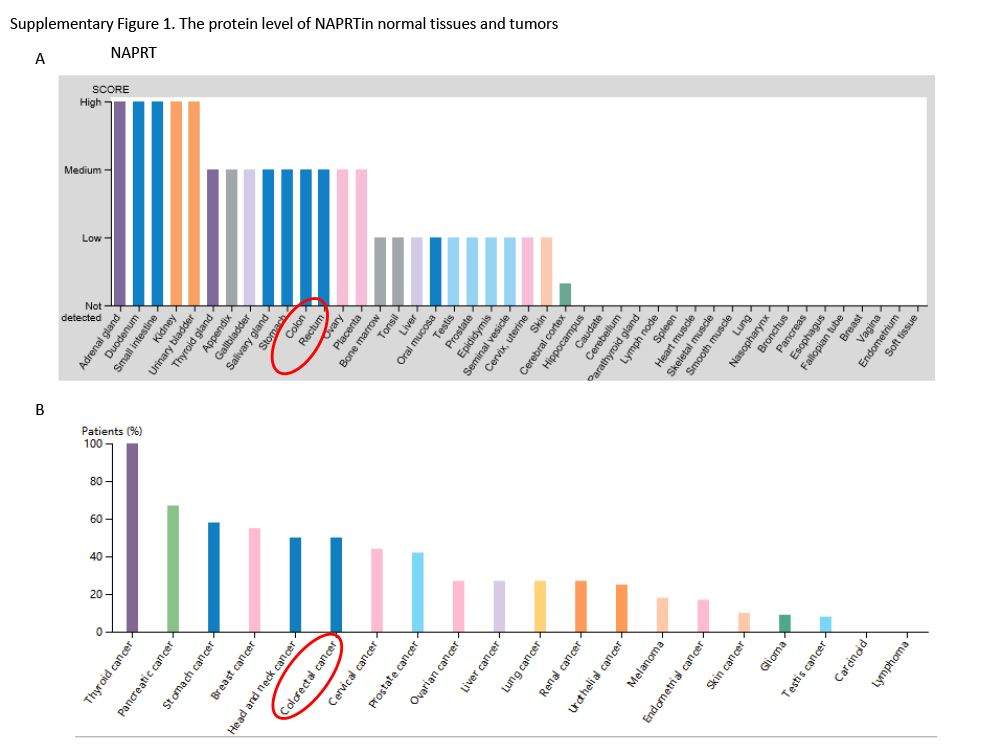

Supplement: Supplementary file 2 [file Image_1.TIF]

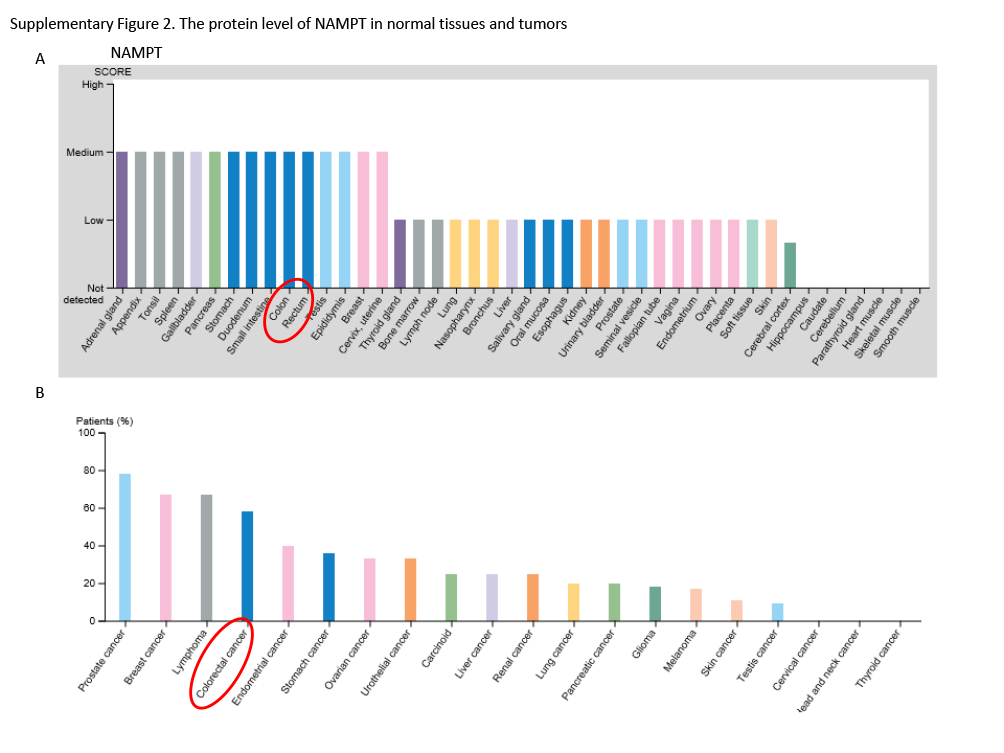

Supplement: Supplementary file 3 [file Image_2.TIF]

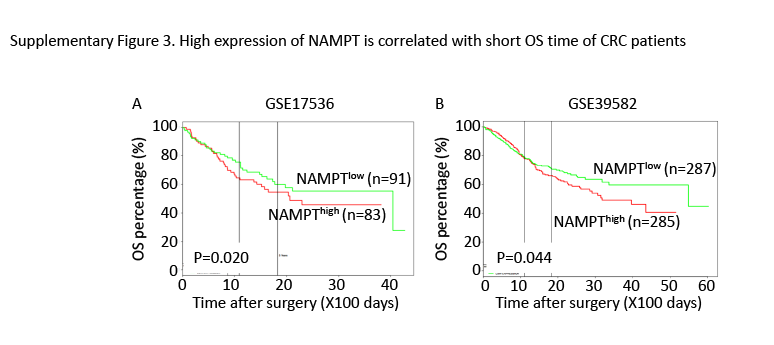

Supplement: Supplementary file 4 [file Image_3.TIF]
